# Effect of Carbon Black on Rutting and Fatigue Performance of Asphalt

**DOI:** 10.3390/ma14092383

**Published:** 2021-05-03

**Authors:** Kunzhi Zhong, Zhi Li, Jianwei Fan, Guangji Xu, Xiaoming Huang

**Affiliations:** 1 School of Transportation, Southeast University, Nanjing 211189, China; kunzhizhong@seu.edu.cn (K.Z.); jianwei_fan@seu.edu.cn (J.F.); guangji_xu@seu.edu.cn (G.X.); 2 CCCC First Highway Consultants Co. Ltd., Xi’an 710075, China; lizhiseu@163.com

**Keywords:** road engineering, carbon black, asphalt modification, anti-deformation performance, anti-fatigue performance

## Abstract

As an additive to improve the performance of asphalt binder, tire pyrolysis carbon black is gradually being used in road engineering, but the effect of carbon black (CB) with different particle sizes on asphalt modification remains to be further studied. In this study, three kinds of particle sizes and three kinds of contents of CB were used to modify asphalt, and different tests were conducted to research the high temperature performance and fatigue resistance of carbon black modified asphalt binder. It is found that the addition of CB can enhance the rutting resistance and medium temperature fatigue performance of virgin asphalt binder in general. However, for CB of 270 μm and 2.6 μm, its addition under certain contents lead to the decrease of high temperature performance and fatigue performance of the asphalt binder. For aged asphalt, the addition of CB decreases the rutting resistance and improves the fatigue resistance. The recommended content and particle size of CB are 2% and 2.6 μm. This study refines the complex effects of CB on asphalt properties, providing a reference for determining the size and content of CB in asphalt modification.

## 1. Introduction

Asphalt pavement is the main pavement form of freeway [1]. In order to cope with the influence of vehicle load, aging and environmental factors, anti-deformation performance and anti-fatigue performance of asphalt pavement should be ensured, and the asphalt properties determine the performance of asphalt mixture applied in pavement [2,3,4]. At present, the main method to enhance asphalt properties is to modify asphalt. There are various kinds of additives, such as polymers [5,6,7], silica gel [8], carbon black [9], carbon nanotube [10], resin [11], polyphosphoric acid [12], wax [13], etc., to modify asphalt according to different needs. Due to the international conscience of society that a more sustainable development is needed, more efficient management of the waste products is being conducted [14,15,16,17]. With regard to the bitumen modifiers, apart from improving the properties of the bitumen, there is an extensive study of the introduction of waste products as bitumen modifiers, to reduce the amount of waste in landfills. Some examples of waste products as bitumen modifiers are ethylene vinyl acetate [18], polyurethane [19,20] and polyethylene [21].

With the increase of vehicle ownership, there are plenty of discarded tires produced each year. There are several ways to deal with these waste tires, including landfilling, retreading, material recovery, energy recovery, etc. Alliotti studied its properties and took the lead in applying CB to asphalt modification [22]. Researches indicated that the addition of CB increased the rutting resistance performance and cracking resistance performance of asphalt binder [23,24], and improved the softening temperature and storage modulus of asphalt [25] and enhanced the strength of the mixture and helped to promote the healing of the mixture [26]. 

At present, the research on CB modified asphalt mainly focuses on two aspects, one is the influence of CB content on asphalt performance, the other is the dispersion of CB in asphalt. However, as a kind of powder additive, it can be speculated that the particle size of CB may also affect the performance of modified asphalt, but related research is not conclusive. Feng studied the effect of CB with two particle sizes, but only under a certain content [27]. Therefore, the influence of CB under different contents and particle sizes on asphalt performance should be further studied.

For this purpose, three kinds of CB with different particle sizes were applied to prepare CB modified asphalt, and the thin film oven test (TFOT) and pressurized aging vessel (PAV) methods were used to age CB modified asphalt. Three main indexes (softening point, penetration and ductility) tests, multiple stress creep recovery (MSCR) test and linear amplitude sweep (LAS) test were used to research the modification effect of CB on asphalt, by which to analyze the rutting resistance and fatigue cracking resistance performances of virgin and aged CB modified asphalt. 

## 2. Materials and Methods

### 2.1. Materials

**Figure 1:**
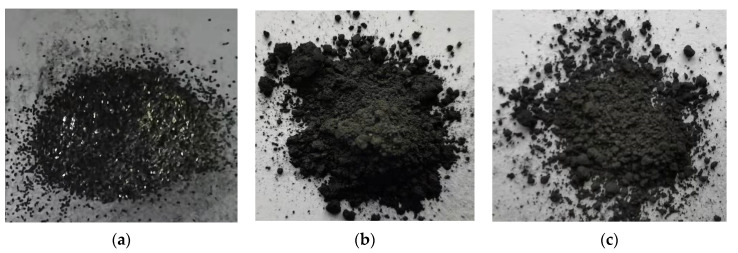
Carbon blacks: (**a**) CB-1, 270 μm, (**b**) CB-2, 25 μm, (**c**) CB-3, 2.6 μm.

In this research, 70# asphalt binder was used, and the penetration of it is 6.4 mm. The properties of asphalt were presented in Table 1. Three types of carbon blacks (CB-1, CB-2 and CB-3) provided by the supplier were used in this study. The CB-1 was a pelleted manufacture with the particle dimension of 270 μm. The particle dimension of CB-2 was about 25 μm, while the particle dimension of CB-3 was about 2.6 μm, as shown in Figure 1.

### 2.2. Preparation of CB Modified Asphalt

Via melt blending, CB modified asphalt binder was made. The neat asphalt binder was heated up until the temperature of it reached to 150 °C and then mixed neat asphalt binder with three different kinds of CBs in different proportions (2%, 4% and 6%), employing a shear mixer at a speed of 3000 r/min. Adding 1/3 mass of carbon black into the asphalt every 10 min, then mixing for 30 min after all carbon black was added, so the whole mixing time was 1h to prepare CB modified asphalt, and referring to previous researches, evenly distributed carbon black modified asphalt can be obtained [24,27,28].

### 2.3. Test Methods

#### 2.3.1. Basic Properties Test

The ductility, softening point and penetration of 10 kinds of asphalt (70# asphalt, CB-1—2%, CB-1—4%, CB-1—6%, CB-2—2%, CB-2—4%, CB-2—6%, CB-3—2%, CB-3—4%, CB-3—6% modified asphalt) were evaluated in accordance with the ASTM standards D113-17 [29], ASTM D5/D5M-20 [30], ASTM D36/D36M-14(2020) [31], respectively. For the ductility test, three duplicates were tested; for the penetration test, one sample was tested; for the softening point test, two duplicates were tested.

#### 2.3.2. Performance Grading Test

The rheological properties of asphalt binder can be estimated by the dynamic shear rheological test (DSR). The rheometer used in this paper is the Anton Paar MCR 102. In this paper, the high temperature grade of asphalt was determined in accordance with the AASHTO M320 [32] method, and the temperature scanning test of asphalt was performed under the controlled strain mode. The scanning temperature was 52 °C, 58 °C, 64 °C, 70 °C, and the scanning frequency was 10 rad/s. The plate employed was 25 mm in diameter, and the gap between parallel plates was 1 mm. For every test, two duplicates were tested.

#### 2.3.3. Multiple Stress Creep Recovery (MSCR) Test

The MSCR test can be employed to investigate the anti-rutting performance of asphalt [33], and the test method was according to ASTM D7405-20 method [34], and for each test, two duplicates were tested. The MSCR test was performed under high temperature grade of asphalt binder. Firstly, the specimen was loaded at a constant creep stress for 1 s length of time creep and followed with a zero-stress recovery of 9 s length of time. Secondly, 20 creep and recovery cycles were performed at a creep stress of 0.1 kPa. The first 10 cycles were for conditioning the specimen. The second ten cycles were designated as cycles N = 1 to 10 and were employed for the collection of data and analysis. Then, 10 creep and recovery cycles were performed at a creep stress of 3.2 kPa. The non-recoverable creep compliance measured at 3.2 kPa (J_nr_3.2) was employed as an assessment of the endurance of bitumen to permanent distortion under repeated loading state, and smaller J_nr_3.2 value represents better rutting resistance. 

#### 2.3.4. Linear Amplitude Sweep (LAS) Test

The LAS test can be employed to evaluate the anti-fatigue performance of the asphalt binder [35], and for each test, two duplicates were tested. LAS test includes two steps. The first step is frequency scanning, i.e., frequency scanning at 0.1% strain in the frequency range of 0.1-30Hz to determine parameters α and B in Equation (1). The second step is linear amplitude scanning, a round of oscillatory load cycles with linearly increasing amplitudes (from 0.1 percent to 30 percent) was conducted at a changeless frequency (10 Hz) to generate accelerated fatigue damage. The viscoelastic continuous damage theory VECD (viscoelastic continuous damage) was used to determine the parameter A_35_ in Equation (1). The test method was according to AASHTO TP 101-12 [36], and larger N_f_ means better fatigue resistance. The asphalt fatigue performance parameter N_f_ was computed by Equation (1):

(1) $$\begin{array}{c} N_{f}=A_{35}{\left(\gamma_{\max}\right)}^{-B} \end{array}$$

where γ_max_ is the maximum expected asphalt strain for a given pavement structure, percent; B is equal to 2α, no unit; N_f_ means loading cycles to failure.

#### 2.3.5. Aging Procedures

The thin film oven test (TFOT) was performed to evaluate the short-term thermal-oxidative aging of CB modified asphalt in accordance with the ASTM D1754 [37], the aging was conducted at 163 °C for 5 h. The long-term thermal-oxidative aging of CB modified bitumen was carried out by the pressurized aging vessel (PAV) test in accordance with the ASTM D6521 [38], the asphalt was aged at 100 °C under 2.1 MPa for 20 h of air pressure.

## 3. Results and Discussion

### 3.1. Penetration, Softening Point and Ductility

**Figure 2:**
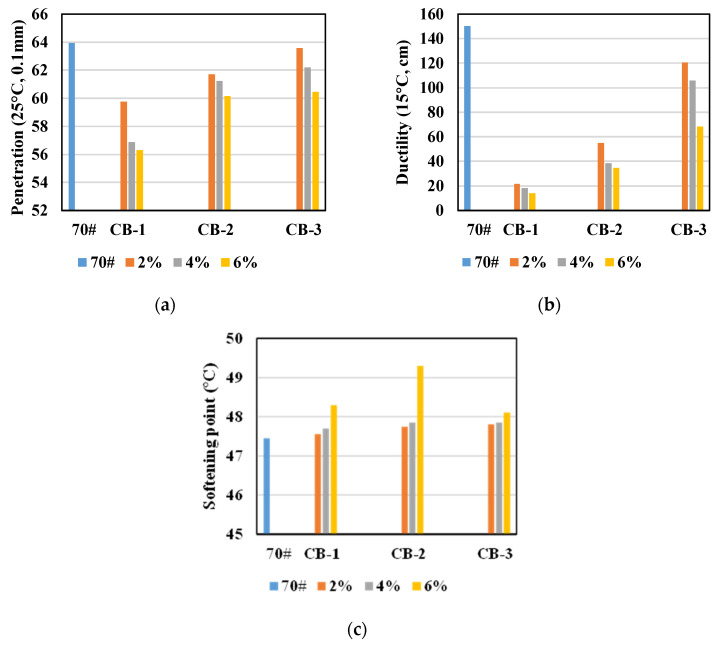
Physical properties of asphalt: (**a**) penetration; (**b**) ductility; (**c**) softening point.

Ductility, penetration and softening point tests of 10 types of asphalt binder specimens were tested, and the test results are shown in Figure 2.

From Figure 2, the following conclusions can be drawn:

The application of CB improves the softening point and reduces the ductility and penetration of asphalt, and this effect is more obvious with the rise of CB dosage. The penetration and ductility of CB modified asphalt diminish after adding CB, and the decrease effect is more obvious with higher CB content. With the addition of CB, the softening point of CB modified asphalt binder was improved, and more added CB means more increasing effect. It indicates that the addition of CB enhances the consistency of asphalt and reduces the flexibility of asphalt, which may be due to the adsorption of some light components in asphalt by CB particles, resulting in the increase of consistency and the decrease of flexibility of asphalt [39,40].

The modification effect of CB with different particle sizes on neat asphalt is different. With the decrease of CB particle size, the effect of CB on penetration and ductility decreases, while the effect on softening point increases, but the effect of 25 μm CB with the content of 6% on softening point is the best.

### 3.2. Rutting Resistance

**Figure 3:**
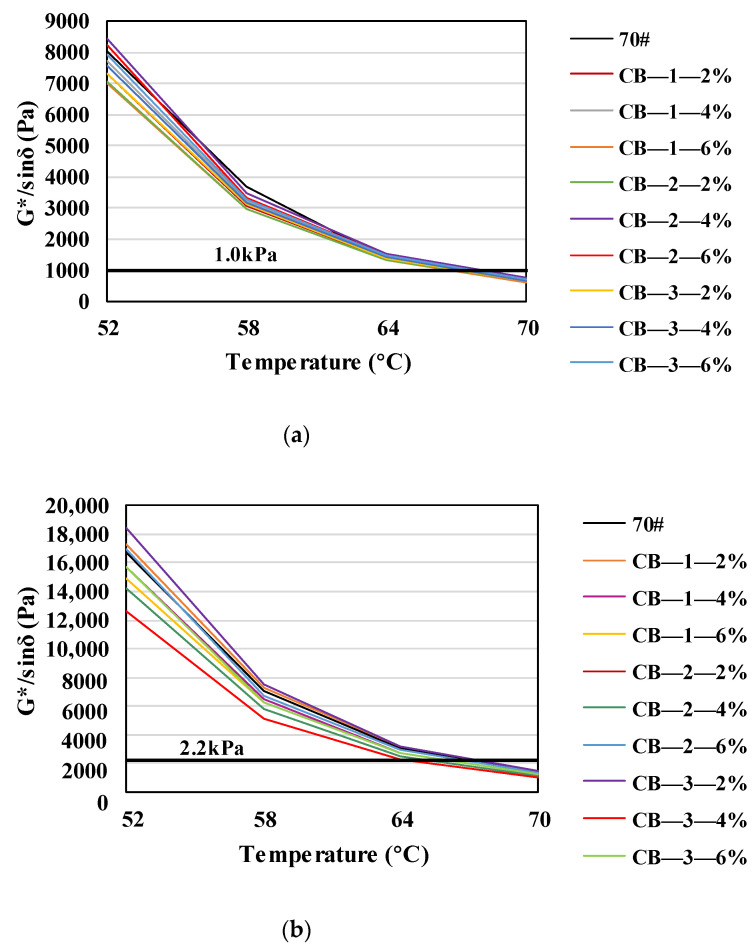
Results of temperature scanning tests: (**a**) virgin asphalt; (**b**) asphalt after TFOT aging.

Through the temperature scanning test of asphalt specimens, the high temperature grade of the asphalt binder was determined. The results are shown in Figure 3.

**Figure 4:**
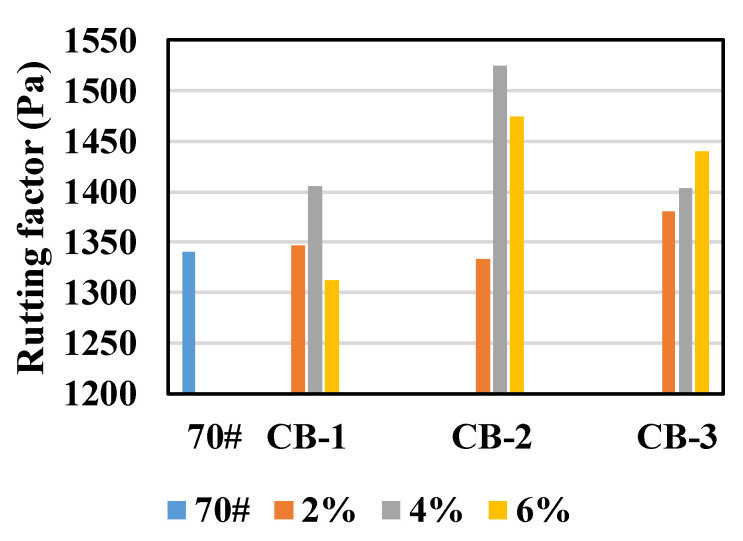
Rutting factor of unaged asphalt specimens.

It can be seen from Figure 3 that for all 10 asphalt specimens, the PG high temperature grade is 64 °C. In Superpave specification, the rutting factor G*/sinδ represents the anti-deformation performance of asphalt binder, and the rutting factors of 10 unaged asphalt specimens at 64 °C were compared, as shown in Figure 4.

It can be seen from Figure 4 that, in general, the rutting factor of asphalt increases after adding CB, indicating that CB is conducive to enhancing the high temperature performance of the asphalt binder, which corresponds to the results of the softening point test. However, the influence of CB content on the rutting factor is different. For 25 μm and 270 μm CB, modified asphalt specimens with 4% carbon black content reach the largest rutting factor, while for 2.6 μm CB, 6% CB content brings the largest rutting factor. The effect of different particle sizes of CB on rutting factor is also different. The improvement effect of 270 μm CB on asphalt rutting factor is the worst, and that of 25 μm CB is the best. With the increase of 2.6 μm CB content, the asphalt rutting factor also increases.

**Figure 5:**
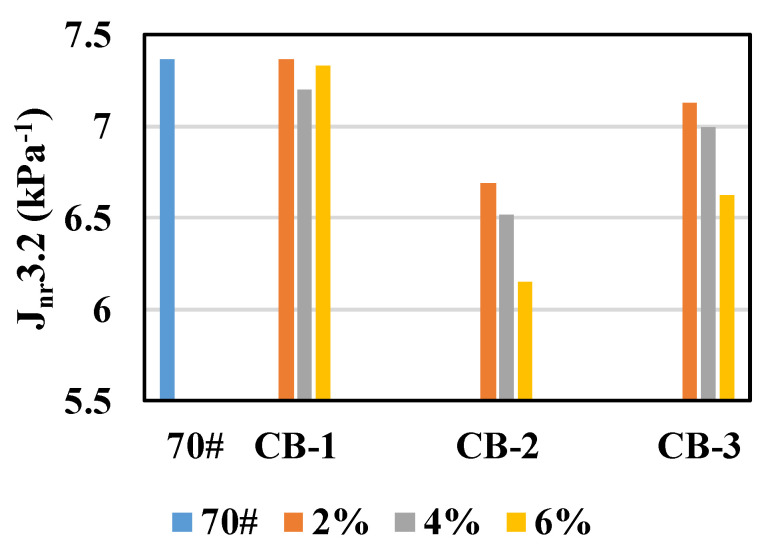
J_nr_3.2 values of unaged asphalt specimens.

MSCR test correlates well with anti-rutting and provides a better correlation to anti-rutting when compared to G*/sinδ, so it is presently being regarded as a substitute for the Superpave high temperature performance criteria G*/sinδ [41]. So as to further investigate the anti-rutting performance of CB asphalt binder, 10 kinds of CB asphalt specimens were tested by MSCR test at a high temperature grade of 64 °C, according to the specification [34]. The non-recoverable creep compliance tested at 3.2 kPa (J_nr_3.2) of specimens are shown in Figure 5.

As seen in Figure 5, J_nr_3.2 values of asphalt decrease after CB added, indicating that CB is beneficial to enhance the anti-rutting performance of the asphalt binder, which corresponds to the results of the softening point test and rutting factor test. However, the effect of CB content on J_nr_3.2 is different. For 2.6 μm and 25 μm CB, 6% of CB content has the smallest effect on J_nr_3.2, while for 270 μm CB, 4% of CB content has the smallest effect on J_nr_3.2. The reason may be that the particle size of 2.6 μm and 25 μm CB is smaller and more evenly distributed in the asphalt, so the effect of improving J_nr_3.2 is obvious, while the particle size of 270 μm CB is larger. When the dosage of CB increases to 6%, the distribution of CB in asphalt is not uniform enough, leading to the decrease of J_nr_3.2 [42].

The effect of different particle sizes of CB on J_nr_3.2 is also different. The improvement effect of 25 μm CB on asphalt J_nr_3.2 is the best, and the improvement effect of 2.6 μm CB on asphalt J_nr_3.2 is the second. The addition of 2.6 μm and 25 μm CB increases, and asphalt J_nr_3.2 also decreases. The improvement effect of 270 μm CB on asphalt J_nr_3.2 is the worst, and the effect of addition is not obvious. The reason may be that CB absorbs light components in asphalt and forms cross-linking structure at the same time. The particle size of 270 μm CB is coarse, and its specific surface area is small, so the effect on J_nr_3.2 is not obvious. The 25 μm CB absorbs light components in asphalt, it forms well cross-linking structure, so J_nr_3.2 decreases obviously. The 2.6 μm CB has smaller particle size and larger specific surface area, it has a better cross-linking structure but absorbs too many light components, so its J_nr_3.2 is larger than that of 25 μm CB modified asphalt [39,40].

### 3.3. Fatigue Resistance

**Figure 6:**
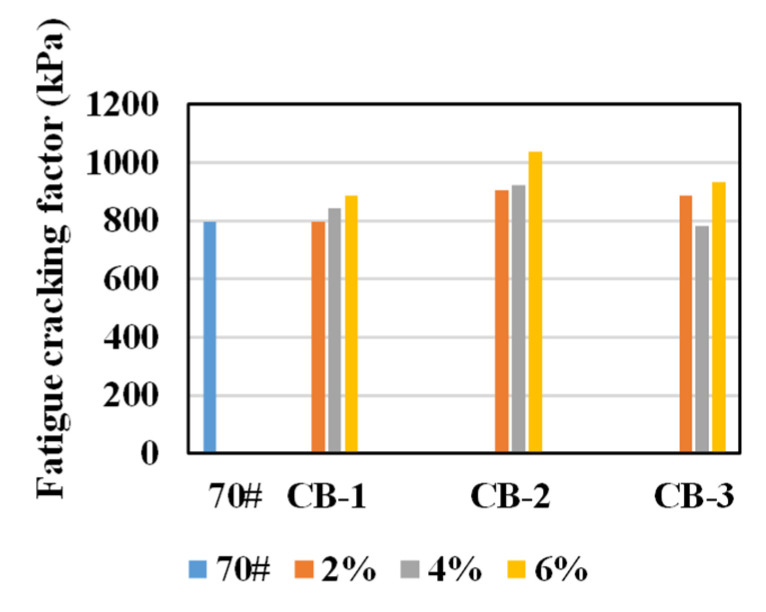
Fatigue cracking factors of unaged asphalt specimens.

Through DSR test of asphalt with loading speed of 10 rad/s at 25 °C, the fatigue cracking factors G* × sinδ of unaged asphalt specimens were obtained, as shown in Figure 6.

It could be found from Figure 6 that, in general, the fatigue factor of asphalt binder increases after adding CB, and the influence of CB content on the fatigue factor is different. For 25 μm and 270 μm CB, the fatigue factor increases with the increase of CB content. For 2.6 μm CB, although the fatigue factor reaches the largest value at 6% of CB content, the fatigue factor is the smallest at 4% of CB content. The effect of CB with different particle sizes on the fatigue factor is also different. The 270 μm CB has the least effect on the fatigue factor of asphalt, while 25 μm CB has the most effect.

Studies have stated that the fatigue cracking factor is inaccurate to describe fatigue resistance of asphalt [43,44]. Therefore, LAS test at 25 °C under 5% maximum strain was applied, and the fatigue performance parameter N_f_ was obtained to represent the fatigue life of asphalt.

**Figure 7:**
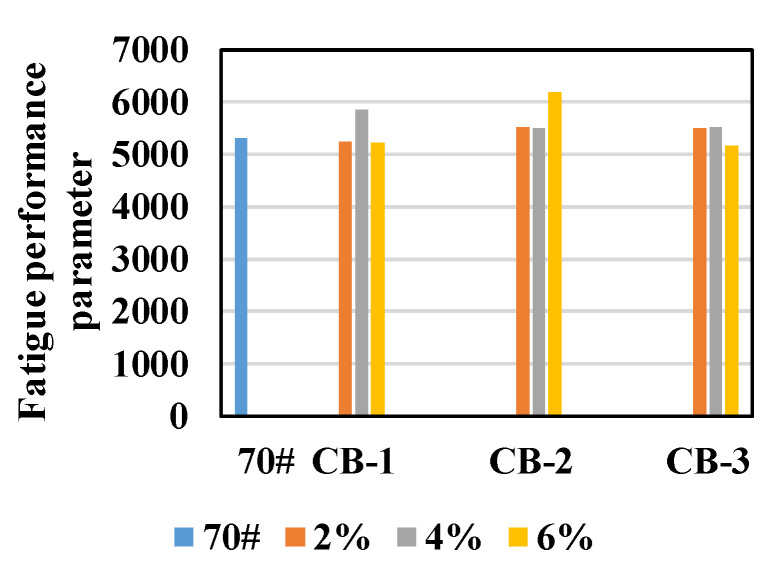
Fatigue performance parameters of unaged asphalt specimens.

From Figure 7, the addition of CB is generally beneficial to enhance the anti-fatigue performance of asphalt binder. For 270 μm CB, the fatigue life of unaged modified asphalt first increases and then decreases with the increase of CB dosage, and the maximum N_f_ value occurs when the CB content is 4%. For 25 μm CB, the fatigue life of 2% and 4% CB content is similar, and the fatigue life of 6% CB content is significantly improved. For 2.6 μm CB, the fatigue life of 2% and 4% CB content is similar, however, the fatigue life decreases when the CB content is 6%.

The effect of 25 μm CB on fatigue life of modified asphalt is the best, and that of 2.6 μm CB is the least. The reason may be that the particle size of 270 μm CB is relatively coarse. When the content reaches 6%, the distribution of CB is not uniform enough, which leads to the decrease of fatigue life. The particle size of 25 μm CB is moderate, which forms a good cross-linking structure when it is evenly distributed in asphalt. Therefore, when the content reaches 6%, the fatigue life increases obviously. The particle size of 2.6 μm CB is smaller, and the specific surface area is larger, which can absorb lighter components. When its content reaches 6%, the fatigue life decreases because the light components are absorbed too much [42].

### 3.4. Aging Performance

The rutting and fatigue resistance of unaged asphalt was studied, its aging performance also needs to be paid great attention. Therefore, the asphalt specimens were aged by TFOT and PAV to further test the influence of CB on asphalt binder aging performance. So as to test the influence of aging on anti-rutting performance of CB modified asphalt binder, the asphalt was aged by TFOT, and the rutting factor at 64 °C and J_nr_3.2 values were tested.

**Figure 8:**
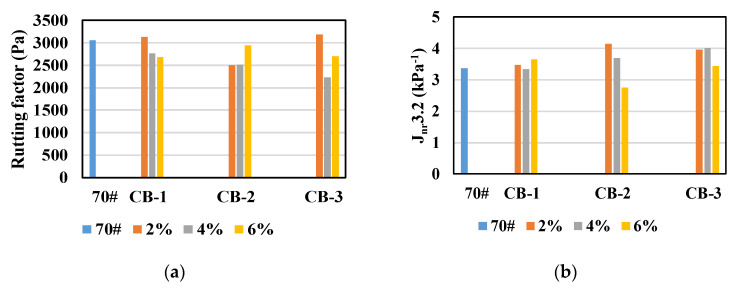
Rutting factors and J_nr_3.2 values of asphalt specimens after TFOT aging: (**a**) rutting factor; (**b**) J_nr_3.2.

**Figure 9:**
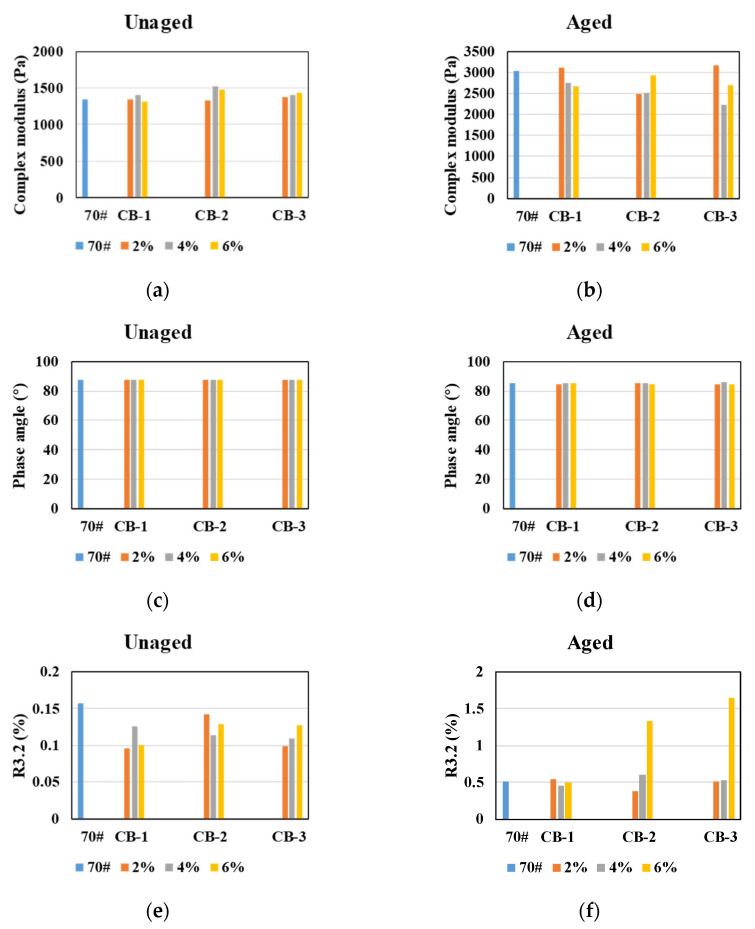
Comparison of unaged asphalt and asphalt after TFOT aging: (**a**,**c**,**e**) unaged asphalt; (**b**,**d**,**f**) asphalt after TFOT aging.

As seen in Figure 8, after TFOT, the rutting factors of CB modified asphalt are generally less than that of neat asphalt, and J_nr_3.2 values are generally greater than that of neat asphalt, indicating that after TFOT aging, the rutting resistance of CB modified asphalt binder is worse than that of neat asphalt binder. This result is contrary to that of unaged asphalt specimens. To analyze the reason, the complex modulus and phase angle of 64 °C temperature scanning test before and after TFOT aging, and R3.2 values of the MSCR test were compared, as shown in Figure 9.

It could be found from Figure 9 that before TFOT aging, overall, the complex modulus of CB modified asphalt binder is larger than that of neat asphalt binder, the phase angle is basically the same as that of neat asphalt binder, and R3.2 is smaller than that of neat asphalt. It shows that the addition of CB makes asphalt stiffer, but its resilience decreases. After TFOT aging, on the whole, the complex modulus of CB modified asphalt binder is less than that of neat asphalt binder, and the phase angle is basically the same as that of neat asphalt, so the rutting factor of CB asphalt is less than that of neat asphalt. The R3.2 values of CB modified asphalt binder is larger than that of neat asphalt binder, especially when the content of 2.6 μm and 25 μm CB is 6%, the R3.2 values of CB modified asphalt is much larger than that of neat asphalt, which indicates that the addition of CB enhances the resilience of asphalt after TFOT. The reason may be that CB absorbs the light components in asphalt, so the resilience of CB asphalt before aging is less than that of a neat asphalt binder. Simultaneously, CB absorbs the light components of asphalt binder, resulting in less volatilization of light components in the aging process. Therefore, after TFOT aging, CB asphalt has better resilience than neat asphalt soft [39,40].

So as to test the influence of aging on the anti-fatigue performance of CB asphalt binder, the asphalt was aged by TFOT and PAV, and the fatigue factor and fatigue performance parameter N_f_ at 25 °C were compared.

**Figure 10:**
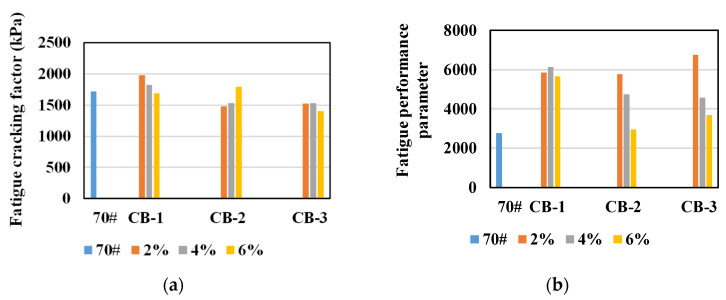
Fatigue cracking factor and fatigue performance parameter of asphalt after PAV aging: (**a**) fatigue cracking factor; (**b**) fatigue performance parameter.

**Figure 11:**
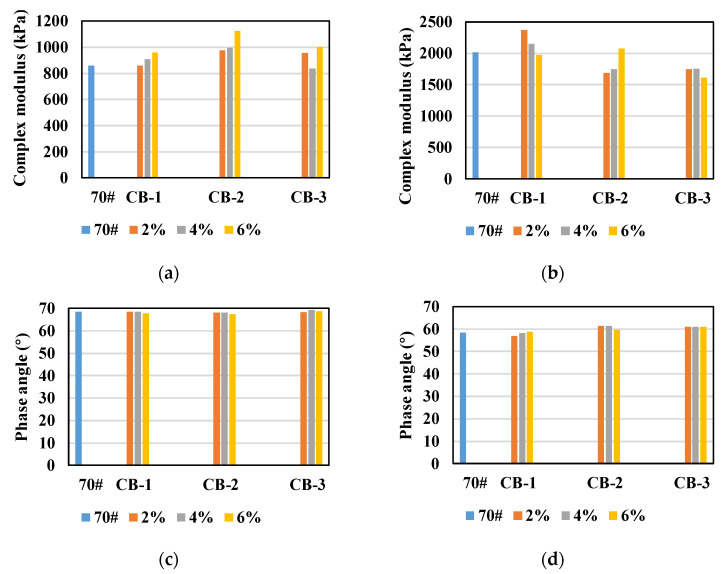
Comparison of unaged asphalt and asphalt after PAV aging: (**a**,**c**) unaged asphalt; (**b**,**d**) asphalt after PAV aging.

It could be found from Figure 10 that, in general, the fatigue factor of CB modified asphalt binder after PAV is less than that of neat asphalt, and N_f_ is greater than that of neat asphalt, indicating that after PAV aging, the fatigue resistance of CB modified asphalt binder is better than that of neat asphalt binder. This result is consistent with that of unaged asphalt, but opposite to that of unaged asphalt. To analyze the reason, the complex modulus and phase angle before and after PAV aging were compared, as shown in Figure 11.

It can be seen from Figure 11 that, before PAV aging, the complex modulus of CB modified asphalt binder is larger than that of neat asphalt binder, and the phase angle is basically the same as that of neat asphalt, which is also the reason why the fatigue factor of CB modified asphalt binder is larger than that of neat asphalt binder. However, it is uncertain whether the addition of CB reduces the fatigue performance of asphalt or hardens the asphalt to increase the fatigue factor. After PAV aging, the complex modulus of CB modified asphalt binder is less than that of neat asphalt binder, and the phase angle is basically the same as that of neat asphalt. Therefore, the fatigue factor of CB asphalt binder is less than that of neat asphalt binder, which indicates that the addition of CB can effectively delay the aging and hardening of asphalt. The reason may be that CB absorbs the light components in the asphalt and prevents the volatilization of the light components in the aging process. Therefore, after PAV aging, the fatigue factor of CB modified asphalt binder is lower than that of neat asphalt binder [39,40].

Based on the above analysis in Section 3.4, the following conclusions can be drawn.

(1) In general, the rutting resistance of CB modified asphalt after TFOT aging is worse than that of neat asphalt binder, which is contrary to the result of unaged asphalt binder. By comparing the changes of complex modulus, phase angle and R3.2 before and after TFOT aging, the main reason is that CB modified asphalt after TFOT aging is softer than neat asphalt. However, not all TFOT aged CB modified asphalt has worse rutting resistance than neat asphalt. For 270 μm CB, when its content is 4%, the rutting resistance of CB modified asphalt binder is better than that of neat asphalt binder, while for 25 μm CB, when the content is 6%, the rutting resistance of CB modified asphalt binder is better than that of neat asphalt binder.

(2) In general, the fatigue resistance of PAV aged CB modified asphalt binder is better than that of neat asphalt binder, which is consistent with the result of unaged asphalt. The effect of CB content on the fatigue resistance of PAV aged CB asphalt is different. For 270 μm CB, the maximum N_f_ is 4%, and for 25 μm and 2.6 μm CB, the maximum N_f_ is 2%. The effect of CB with different particle sizes on the fatigue resistance of asphalt is also different. The 270 μm CB has the best effect on improving the fatigue resistance of asphalt. For 25 μm and 2.6 μm CB, with the increase of CB dosage, the anti-fatigue performance of asphalt binder decreases, but it is still better than that of neat asphalt.

(3) Since the long-term performance of pavement is pretty important, selecting asphalt with the best fatigue performance will help improve the performance of pavement, so the content and particle size of CB that would be recommended are 2% and 2.6 μm.

## 4. Conclusions

In this paper, 270 μm, 25 μm and 2.6 μm CB with the content of 2%, 4% and 6% were added into asphalt to research its effect on rutting and fatigue resistance of asphalt before and after aging. In summary, the following conclusions can be made:

(1) The addition of CB improves the softening point and reduces the ductility and penetration of asphalt, and this effect is more obvious with the increase of CB content. The effects of CB with different particle sizes on softening point, ductility and penetration of asphalt are different. When the particle size of CB becomes finer, the influence of CB on penetration and ductility decreases, and 25 μm CB has the best effect on softening point.

(2) The effects of CB with different particle sizes on rutting resistance of asphalt is different. When the content of 270 μm CB is 2%, the rutting resistance of CB modified asphalt binder is lower than that of neat asphalt binder. For 25 μm and 2.6 μm CB, the rutting resistance of CB increases with the increase of CB dosage.

(3) The effects of CB with different particle sizes on fatigue resistance of asphalt is different. When the content of 270 μm CB is 2% and 6% or 2.6 μm CB content is 6%, the fatigue resistance of CB modified asphalt is lower than that of neat asphalt. When the content of 270 μm CB is 4% or 25 μm CB content is 6%, the fatigue resistance of CB modified asphalt is significantly higher than that of neat asphalt binder.

(4) The rutting resistance after TFOT aging of CB modified asphalt is generally worse than that of neat asphalt. Only when 270 μm CB content is 4% or 25 μm CB content is 6%, the rutting resistance of CB modified asphalt binder is better than that of neat asphalt binder.

(5) CB improves the fatigue resistance of PAV aging asphalt generally. This effect of 270 μm CB on asphalt fatigue resistance is the best, but for 25 μm and 2.6 μm CB, with the increase of CB dosage, the anti-fatigue performance of the asphalt binder decreases, so it is necessary to control the content of CB with different particle sizes.

(6) Combined with all the test results, the content and particle size of CB that would be recommended are 2% and 2.6 μm. Further research could be done by investigating the influence of CB on the performance of the asphalt mixture.

## Figures and Tables

**Table 1:** Properties of 70# asphalt binder.

Indicators	Unit	Values
Softening point	°C	47.5
Ductility	cm	150
Penetration	0.1 mm	64
Flash point	°C	>260
Density	g/cm^3^	1.034

## Data Availability

The data presented in this study are available on request from the corresponding author.
